# Supplementary material for: Manufacturing and Characterization of Highly Environmentally Friendly Sandwich Composites from Polylactide Cores and Flax-Polylactide Faces
Source: Polymers (Basel). 2021 Jan 21;13(3):342. doi: 10.3390/polym13030342 (PMC7865247; doi:10.3390/polym13030342)
Supplement: Supplementary file 1 [file polymers-13-00342-s001.pdf]

polymers

MDPI

Article

Manufacturing and Characterization of High Environmentally Friendly Sandwich Composites from Polylactide Cores and Flax-Polylactide Faces

Diego Lascano *, Rene Guillen-Pineda, Luis Quiles-Carrillo, Juan Ivorra-Martínez *, Rafael Balart, Nestor Montanes and Teodomiro Boronat

[1] Technological Institute of Materials (ITM), Universitat Politècnica de València (UPV), Plaza Ferrándiz y Carbonell 1, 03801 Alcoy, Spain; reguipi@upv.es (R.G.-P.); luiquic1@epsa.upv.es (L.Q.-C.); rbalart@mcm.upv.es (R.B.); nesmonmu@upvnet.upv.es (N.M.); tboronat@dimm.upv.es (T.B.)

* Correspondence: dielas@epsa.upv.es (D.L.); juaivmar@doctor.upv.es (J.I.-M.); Tel.: +34-966-528-433 (D.L.)

Supplementary information

Qualitative assessment of shape memory behaviour of PLA sheets.

The shape memory behaviour of PLA films was qualitatively assessed under dual-shape effect using thermal-activated conditions. For this purpose, PLA sheets with a thickness of 100, 250 and 500 µm were firstly shaped into a semi-hexagonal shape (to simulate the behaviour that honeycomb core would have) by hot-press moulding. To carry out the thermal-activation shape memory test, two cycles were performed (programming and recovery cycle). The switching transition temperature (T_{trans}) was set at 70 °C (10 °C above the T_g, but remarkably below the cold crystallization process); this temperature will determine both the programming and recovery cycle [1-3]. The scheduled cycle was the following. In a first stage, PLA sheets were heated above the corresponding T_{trans}, in a hot press for 2 min (stabilization time) and were allowed to get the semi-hexagonal shape by constant-stress. After that, the semi-hexagonal-shaped sheets were cooled down to room temperature (≈ 25 °C) maintaining the compression stress; the temporary shape was reached when the external stress was released. The recovery cycle was carried out by placing the semi-hexagonal shaped sheets into a pre-heated oven at T_{trans} (70 °C) for 2 min (stabilization time). Finally, the shape memory recovery state was visually observed. At least 3 tests for each film were performed to obtain reliable data.

Citation: Lascano, D.; Guillen-Pineda, R.; Quiles-Carrillo, L.; Ivorra-Martínez, J.; Balart, R.; Montanes, N.; Boronat, T. Manufacturing and Characterization of High Environmentally Friendly Sandwich Composites from Polylactide Cores and Flax-Polylactide Faces. *Polymers* **2021**, *13*, 342. https://doi.org/10.3390/polym13030342

Received: 10 December 2020
Accepted: 20 January 2021
Published: 22 January 2021

Publisher's Note: MDPI stays neutral with regard to jurisdictional claims in published maps and institutional affiliations.

Copyright: © 2021 by the authors. Licensee MDPI, Basel, Switzerland. This article is an open access article distributed under the terms and conditions of the Creative Commons Attribution (CC BY) license (http://creativecommons.org/licenses/by/4.0/).

Results and Discussion

The shape memory behaviour of PLA-based sheets could be interesting for widening the potential use of PLA-based honeycomb cores. Qualitative assessment of the shape memory behaviour of PLA semi-hexagonal sheets was carried out by one-way dual shape effects (DSE). This effect defines that the film can reach only its original shape (flat film) after a temporary shape has been achieved (semi-hexagonal shape) when an external stimulus is triggered. As can be seen in Figure S1, the recovery of the original shape is pushed by the entropic elasticity of the polymer network. In the first stage (0 s) the sheet still has its temporary semi-hexagonal shape. At this stage, it has not been exposed to any external stimulus yet, that would make it recover its original shape. After heating the semi-hexagonal sheets (temporary shape) at the T_{trans} (70 °C), the polymer chains try to return to the rubber-elastic state distinguished by the change of geometry in the films. This phenomenon can be clearly seen after 30 seconds at 70 °C. Obviously, the sheet thickness plays an important role in this recovery. The thinner the thickness, the faster the recovery as can be seen for sheets with 100 and 250 µm. Unlike, the 500 µm sheet that does not offer any change in its geometry (recovery). The recovery for the 500 µm sheets triggers after 90 s at 70 °C. At this recovery time the 100 µm sheet, has practically recovered its original flat shape. After 2 minutes, it is clear that the 100 and 200 µm sheets have recovered their

original flat shape. Unlike the 500 μm sheet may need more exposure time to temperature to fully recover the initial flat shape.

Anyway, it could be observed the excellent shape memory behaviour that PLA offers, which is better for low thickness sheets. PLA is one of the materials that due to its semi-crystalline structure has an excellent shape memory capacity because the combination of both crystalline and amorphous phases. The crystalline phase is responsible for setting the temporary shape while the amorphous phase acts as a switching phase [1]. If PLA shape is changed at high temperatures (above its glass transition temperature), the crystalline domains of PLA have greater mobility. These act as physical cross-linking points, making the acquired temporal shape to be stable (in this case, maintaining the semi-hexagonal shape). On the other hand, at this temperature the recovery capacity of the material is reduced. In this case, increasing the recovery time, this can be attributed to the high-tension energy accumulated by the sheet deformation and the loss of entropy, resulting from the cooling process [4]. In previous studies [5], and some research works as the one carried out by Leonés et al. [6], they reported that the addition of plasticizers such as lactic acid oligomer (OLA) to PLA, helped to improve the shape memory capacity of plasticized PLA by adding up to 20% OLA. They reported a remarkable decrease in recovery time to 10 s. In addition, OLA plasticizer lowers the glass transition temperature T_g. This temperature is usually used as a transition temperature in shape memory studies and can drop to 35 °C. Continuous research is being carried out in the field of PLA shape memory behaviour by blending. Peponi et al. [7] reported the shape memory behaviour of PLA and polycaprolactone (PCL) blends where the PLA acts as the element that fixes the temporary form, and the crystalline PCL works as the switch segment. The excellent ability of the material to both set the temporary shape and return to its original form was observed, presenting recovery percentages above 90%, which could be an interesting feature in 3d-printed materials, packaging, and medical devices [8].

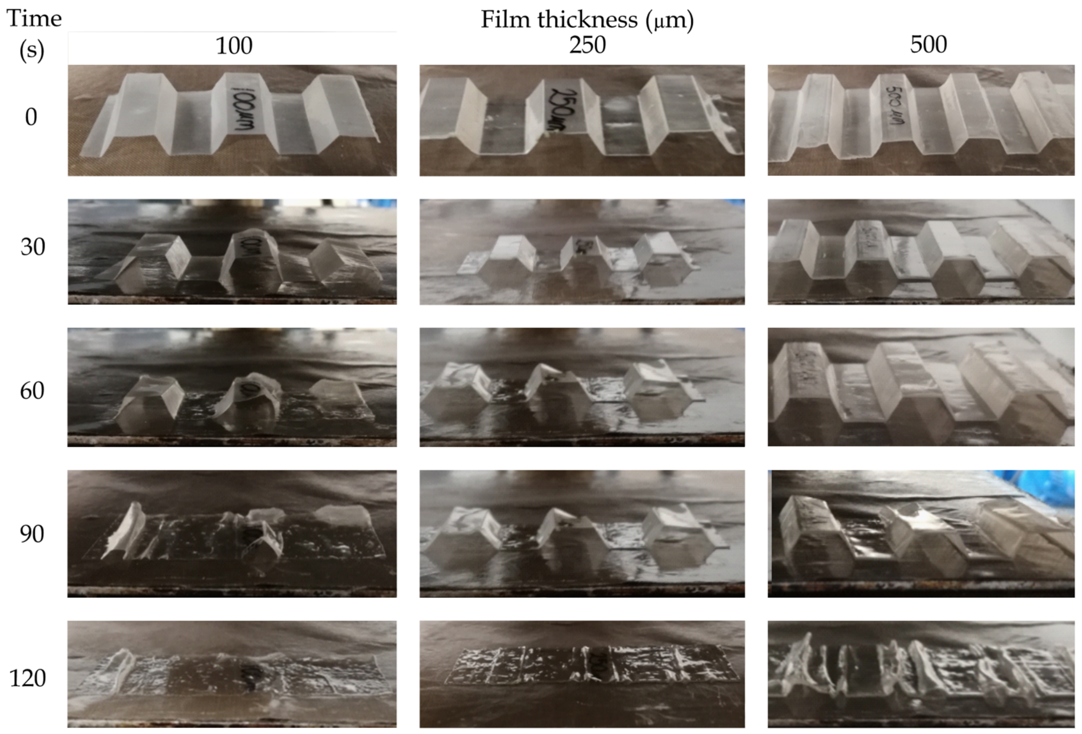

Figure S1. Visual illustration of the qualitative shape memory evaluation in the recovery cycle of PLA films with different thicknesses: 100 μm (left column), 250 μm (middle column), and 500 μm (right column).

In addition, it was possible to observe the excellent shape memory capacity of PLA; since the analyzed films completely recovered their original shape, having a better effect

on the thinner sheets, which translates into shorter recovery times. This opens new possibilities for PLA-based honeycombs since it is possible to tailor the geometry by triggering geometry changes using temperature.

Author Contributions: Conceptualization, R.B. and R.G.-P.; methodology, T.B., D.L and L.Q.-C..; validation, N.M., R.B., T.B and R.G.-P.; formal analysis, D.L. and J.I.-M.; investigation, D.L. and R.G.-P.; data curation, R.G.-P. and L.Q.-C; writing—original draft preparation, D.L and J.I.-M.; writing—review and editing, R.B. and N.M.; supervision, N.M.; project administration, R.B.; funding acquisition, R.B.

Funding: This research work was funded by the Spanish Ministry of Science and Innovation (MICI) project number MAT2017-84909-C2-2-R.

Acknowledgments: D.L. wants to thank Universitat Politècnica de València (UPV) for the grant received through the PAID-01-18 program. L.Q.-C. thanks Generalitat Valenciana (GVA) for his FPI grant (ACIF/2016/182) and the Spanish Ministry of Education, Culture, and Sports (MECD) for his FPU grant (FPU15/03812). R.G.-P. is recipient of a Santiago Grisolía contract (GRISOLIAP/2018/168) from GVA. J.I-M. wants to thank Universitat Politècnica de València for his FPI grant (PAID-2019-SP20190011) and Spanish Ministry of Science, Innovation and Universities for his FPU grant (FPU19/01759).

Conflicts of Interest: The authors declare no conflict of interest.

References

1. Dogan, S.; Boyacioglu, S.; Kodal, M.; Gokce, O.; Ozkoc, G. Thermally induced shape memory behavior, enzymatic degradation and biocompatibility of PLA/TPU blends:"Effects of compatibilization". *J. Mech. Behav. Biomed. Mater.* **2017**, *71*, 349–361.
2. Cai, S.; Sun, Y.-C.; Ren, J.; Naguib, H.E. Toward the low actuation temperature of flexible shape memory polymer composites with room temperature deformability via induced plasticizing effect. *J Mater. Chem. B.* **2017**, *5*, 8845–8853.
3. Berg, G.J.; McBride, M.K.; Wang, C.; Bowman, C.N. New directions in the chemistry of shape memory polymers. *Polymer* **2014**, *55*, 5849–5872.
4. Lai, S.-M.; Lan, Y.-C. Shape memory properties of melt-blended polylactic acid (PLA)/thermoplastic polyurethane (TPU) bio-based blends. *J. Polym. Res.* **2013**, *20*, 140.
5. Lascano, D.; Moraga, G.; Ivorra-Martinez, J.; Rojas-Lema, S.; Torres-Giner, S.; Balart, R.; Boronat, T.; Quiles-Carrillo, L. Development of Injection-Molded Polylactide Pieces with High Toughness by the Addition of Lactic Acid Oligomer and Characterization of Their Shape Memory Behavior. *Polymers* **2019**, *11*, 2099.
6. Leonés, A.; Sonseca, A.; López, D.; Fiori, S.; Peponi, L. Shape memory effect on electrospun PLA-based fibers tailoring their thermal response. *Eur. Polym. J.* **2019**, *117*, 217–226.
7. Peponi, L.; Sessini, V.; Arrieta, M.P.; Navarro-Baena, I.; Sonseca, A.; Dominici, F.; Gimenez, E.; Torre, L.; Tercjak, A.; López, D. Thermally-activated shape memory effect on biodegradable nanocomposites based on PLA/PCL blend reinforced with hydroxyapatite. *Polym. Degrad. Stab.* **2018**, *151*, 36–51.
8. Sonseca, A.; Madani, S.; Munoz-Bonilla, A.; Fernandez-Garcia, M.; Peponi, L.; Leones, A.; Rodriguez, G.; Echeverria, C.; Lopez, D. Biodegradable and Antimicrobial PLA-OLA Blends Containing Chitosan-Mediated Silver Nanoparticles with Shape Memory Properties for Potential Medical Applications. *Nanomaterials* **2020**, *10*, doi:10.3390/nano10061065.
